# Integrative proteome and metabolome unveil the central role of IAA alteration in axillary bud development following topping in tobacco

**DOI:** 10.1038/s41598-024-66136-4

**Published:** 2024-07-03

**Authors:** Mingmin Zou, Dandan Zhang, Yixuan Liu, Zepeng Chen, Tingyu Xu, Zhuwen Ma, Jiqin Li, Wenji Zhang, Zhenrui Huang, Xiaoying Pan

**Affiliations:** 1 Guangdong Key Laboratory for Crops Genetic Improvement, Guangdong Provincial Engineering and Technology Research Center for Tobacco Breeding and Comprehensive Utilization, Crops Research Institute, Guangdong Academy of Agricultural Sciences (GAAS), Guangzhou, 510640 China; 2 China National Tobacco Corporation, Guangzhou, 510610 Guangdong Province China

**Keywords:** Axillary bud, Proteome and metabolome, IAA, ROS, Tobacco, Proteomics, Plant development, Metabolism

## Abstract

Axillary bud is an important aspect of plant morphology, contributing to the final tobacco yield. However, the mechanisms of axillary bud development in tobacco remain largely unknown. To investigate this aspect of tobacco biology, the metabolome and proteome of the axillary buds before and after topping were compared. A total of 569 metabolites were differentially abundant before and 1, 3, and 5 days after topping. KEGG analyses further revealed that the axillary bud was characterized by a striking enrichment of metabolites involved in flavonoid metabolism, suggesting a strong flavonoid biosynthesis activity in the tobacco axillary bud after topping. Additionally, 9035 differentially expressed proteins (DEPs) were identified before and 1, 3, and 5 days after topping. Subsequent GO and KEGG analyses revealed that the DEPs in the axillary bud were enriched in oxidative stress, hormone signal transduction, MAPK signaling pathway, and starch and sucrose metabolism. The integrated proteome and metabolome analysis revealed that the indole-3-acetic acid (IAA) alteration in buds control dormancy release and sustained growth of axillary bud by regulating proteins involved in carbohydrate metabolism, amino acid metabolism, and lipid metabolism. Notably, the proteins related to reactive oxygen species (ROS) scavenging and flavonoid biosynthesis were strongly negatively correlated with IAA content. These findings shed light on a critical role of IAA alteration in regulating axillary bud outgrowth, and implied a potential crosstalk among IAA alteration, ROS homeostasis, and flavonoid biosynthesis in tobacco axillary bud under topping stress, which could improve our understanding of the IAA alteration in axillary bud as an important regulator of axillary bud development.

## Introduction

Tobacco (*Nicotiana tabacum* L.) is an economically important commercial crop, with the leaf as the primary product^1^. Topping, the decapitation or removal of the apical bud, is necessary to enhance tobacco leaf development or maturation before harvesting. However, axillary shoot outgrowth induced by topping is undesirable since it reallocates resources to axillary buds, reducing the yield and quality of the main leaves^2–5^. Axillary shoot outgrowth can be inhibited by manually removing the suckers and applying fatty alcohols, flumetralin, or maleic hydrazide. However, these methods are time-consuming and labor-intensive. Besides, the chemicals may persist after leaf processing due to environmental variability^6^. Therefore, controlling tobacco apical and axillary bud development after topping is a crucial research focus in tobacco farming. Notably, molecular biology-focused research may provide alternate ways to control the axillary bud growth after topping in tobacco.

Axillary bud development is regulated by a complex mechanism involving various endogenous and environmental factors, such as phytohormones, nutrients, and light. IAA is one of the classic phytohormones, which is transported basipetally through the stem but does not enter buds and therefore acts indirectly to regulate bud growth^7^. Several studies have demonstrated cytokinin (CTK) and strigolactone (SL) are possible second messenger for IAA signaling in regulating axillary bud activity. The apically-derived IAA transported in the stem positively regulates the expression of strigolactone biosynthesis genes and down-regulates cytokinin levels, which inhibits and induces bud outgrowth, respectively^8,9^. In addition, the IAA alteration in buds is required for the development of axillary bud, and hormones, flavonoids and sugars can control IAA level by regulating IAA synthesis and transport in buds^10–12^. To sum up, the axillary bud outgrowth induced by topping is inseparable from the alteration of IAA in stem and buds. However, it’s unclear that how the IAA integral status in buds affect axillary buds development.

High-throughput and large-scale studies on tobacco have revealed the molecular mechanisms underlying axillary bud growth in response to topping. For example, a previous study identified 179 significantly expreesed genes following topping by RNA-seq. Among them, the genes related to wounding, phytohormone metabolism, and secondary metabolite biosynthesis were significantly up-regulated after topping and down-regulated after suckercide treatments^13^. In another study, most differentially expressed genes after topping were involved in starch and sucrose metabolism, glycolysis/gluconeogenesis, pyruvate metabolism, plant hormone signal transduction, and other processes in the axillary bud^14^. Additionally, secondary metabolism, hormone metabolism, signaling/transcription, stress/defense, protein metabolism, and carbon metabolism were identified before and after topping using a combination of suppression subtractive hybridization and miRNA deep sequencing^15^. Based on dynamic IncRNA-seq, topping promotes axillary bud development due to the altered expression levels of IncRNAs associated with hormone signal transduction and glycometabolism, compared to untopped plants^16^. These reports imply that topping can cause significant changes of metabolites in axillary buds. Recent studies have increasingly demonstrated that metabolite alteration are also required for growth and developmental regulation^10,17^. However, there has not yet been a comprehensive analysis of the metabolites profile in the axillary buds.

In this study, we provide a proteomic and metabolic signature of the axillary buds in untopped and topped tobacco. Such reports could link the expression levels of proteins and the accumulation of metabolites to the mechanisms regulating axillary bud development. The temporal dynamics of protein abundance and metabolite accumulation profiles in the axillary buds were described. Moreover, an integrative analysis of proteome and metabolome was performed, emphasizing related metabolites and proteins in plant hormone signal transduction, ROS homeostasis, and antioxidants. The findings from this study will improve our understanding of the mechanisms responsible for axillary bud growth in tobacco.

## Results

### Outgrowth of axillary buds in response to topping

**Figure 1:**
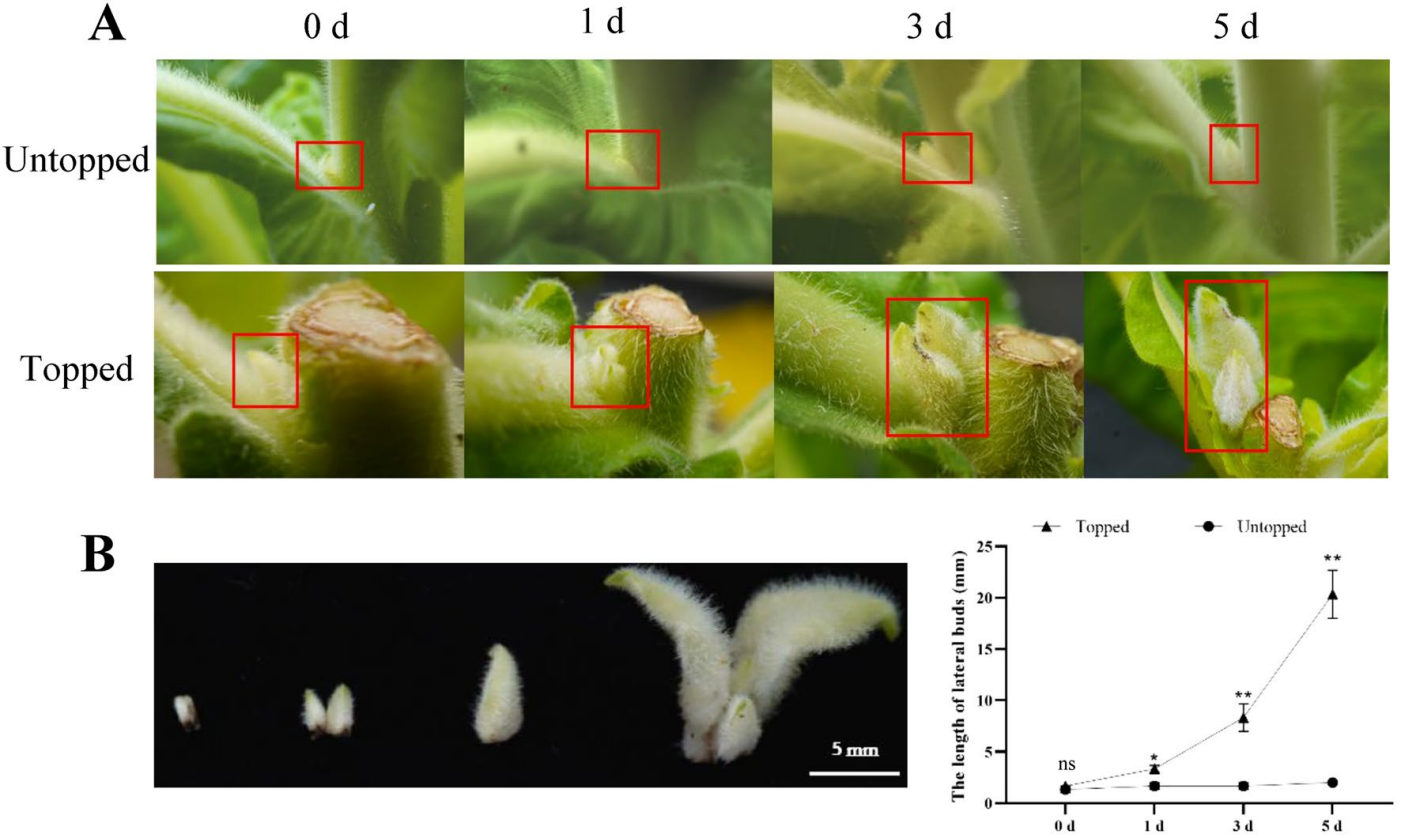
Axillary buds outgrowth of untopped and topped tobacco plants after 1, 3 and 5 days. (**A**) Phenotypes of axillary buds in different time points after topping. Axillary buds were showed as red box. (**B**) The length of lateral buds in topped and untopped plants. The data shown are two-tail *t*-test: * *P* < 0.05; ** *P* < 0.01; ns *P* > 0.05.

We compared the growth of axillary buds in response to untopping and topping in tobacco (Fig. 1A). The growth of the axillary buds was distinctly accelerated after topping, as the length of axillary buds increased about 2, 5, 12 times at 1, 3, 5 days after topping, respectively (Fig. 1B). In contrast, the length of axillary buds showed no significant change within 5 days in untopped tobacco plants (Fig. 1C). It means that topping can significantly accelerate the growth of axillary buds in tobacco. To explore the metabolomic and proteomic changes of axillary buds in response to topping in tobacco, twelve libraries of the axillary buds from untopping and topping plants were constructed for omics analysis.

### UPLC-MS/MS identification of DAMs associated with axillary bud metabolome following topping

**Figure 2:**
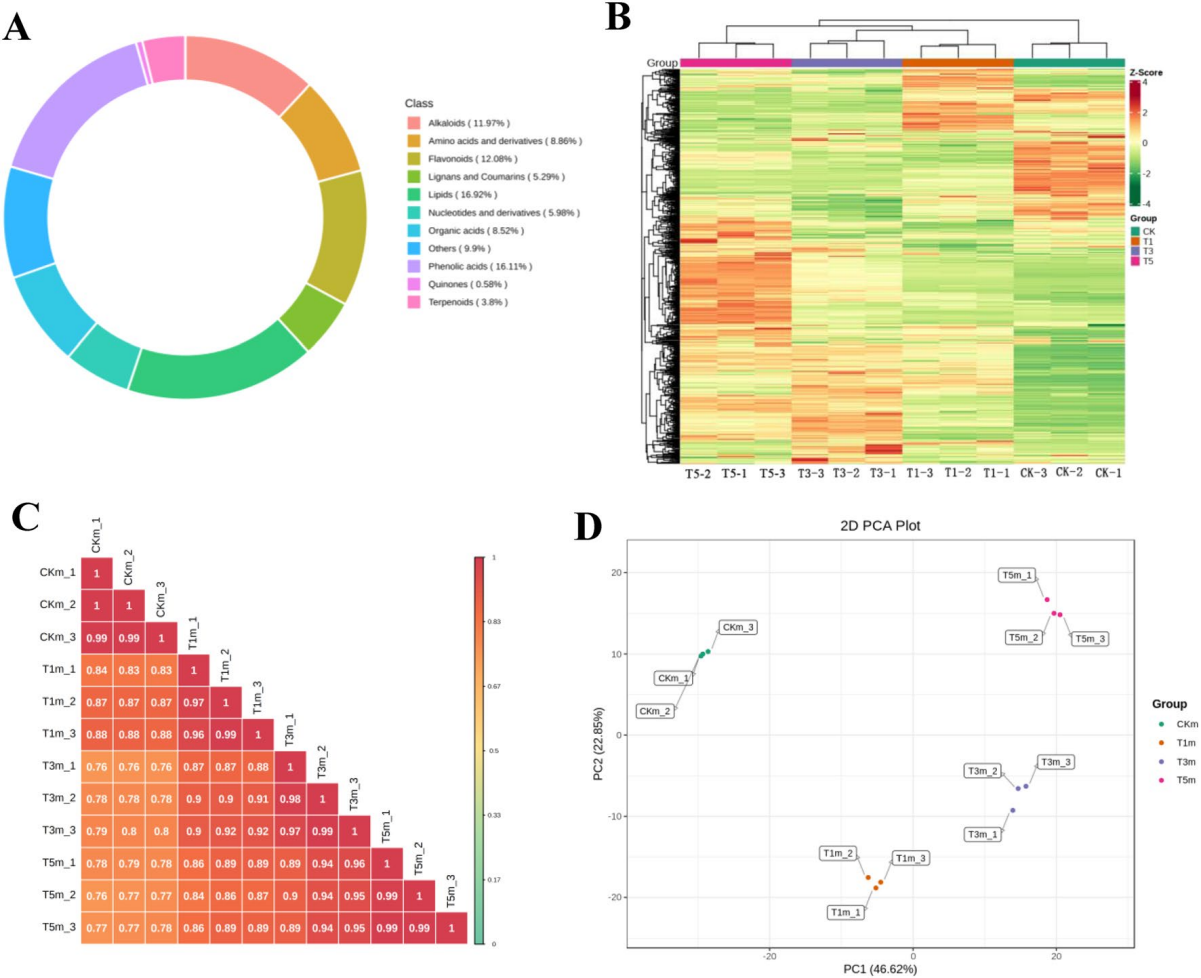
Qualitative and quantitative analysis of the metabolomics data. (**A**) Classification of metabolites with annotated structures. (**B**) Hierarchical cluster analysis of metabolite profiles. (**C**) Heat map of samples obtained from correlation analysis. (**D**) PCA analysis plots of the four group samples.

The widely targeted metabolomic analysis of axillary buds in untopped and topped tobacco plants at 1 (T1), 3 (T3), and 5 (T5) days after topping, detected 869 metabolites across all the samples. The detected metabolites were divided into 11 categories. Most of these metabolites were primary lipids (147, 16.92%), phenolic acids (140, 16.11%), flavonoids (105, 12.08%), and alkaloids (104, 11.97%). Interestingly, only five quinones were detected (Fig. 2A). Based on the cluster and correlation analysis, the metabolites in the 12 samples were divided into four groups (Fig. 2B) with significant intragroup correlations (Fig. 2C). Additionally, PCA revealed significant differences among the four groups (Fig. 2D). PC1 and PC2 accounted for 46.62 and 22.85% of the samples, respectively, with a cumulative contribution rate of 69.47%. On the PC1 axis, the control (CK) and T1 samples significantly differed from the T3 and T5 samples, implying metabolite differences between the axillary meristem initiation and axillary bud growth.

**Figure 3:**
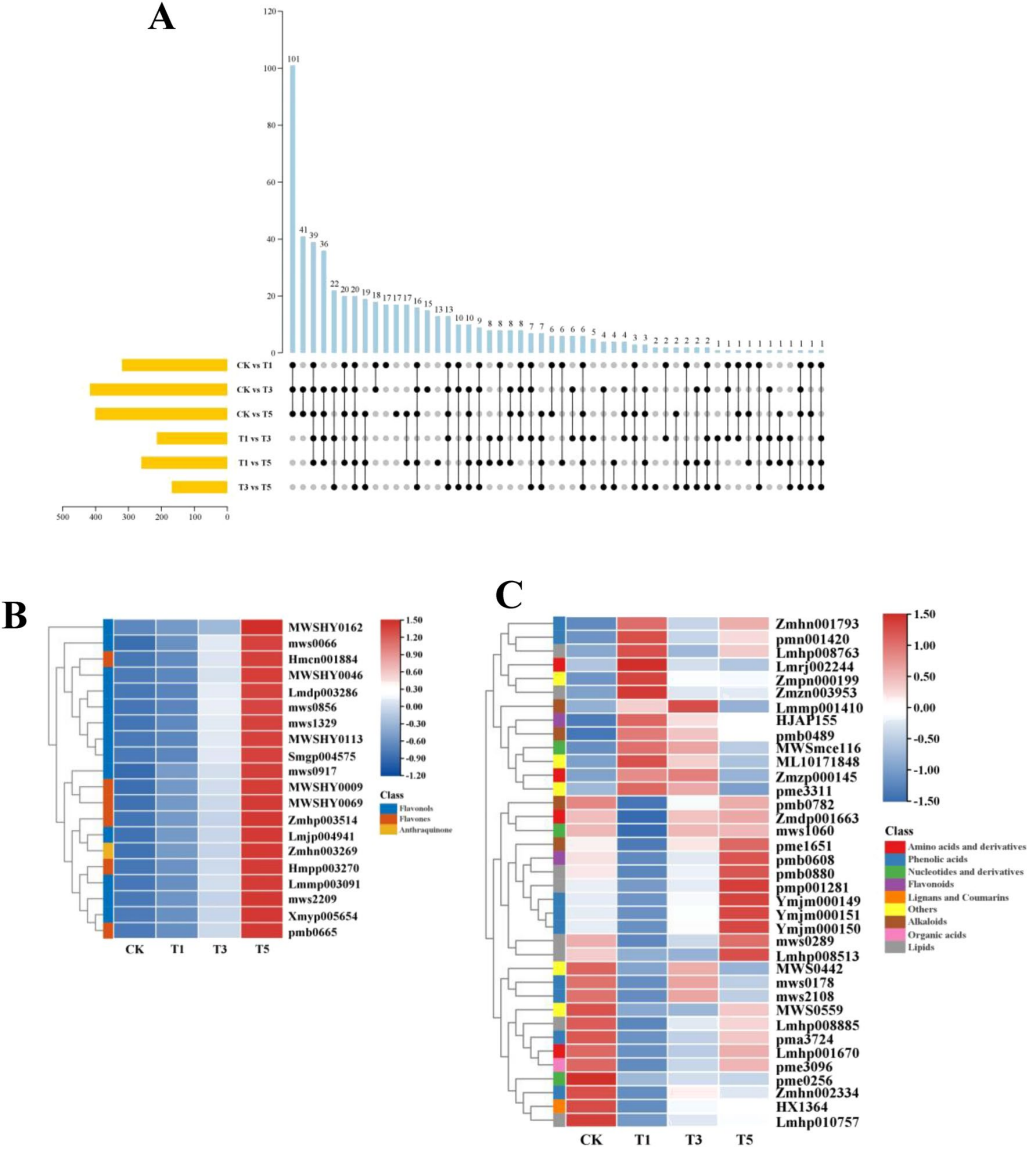
Identification and characterization of DAMs. (**A**) Advanced Venn diagram and Upset plots showing the DAMs in different comparison groups. (**B**) Accumulation profiles of 20 DAMs common in six comparison groups. (**C**) Accumulation profiles of 37 DAMs specific to T1 vs. CK group.

Moreover, 569 differentially accumulated metabolites (DAMs) were identified from all comparison groups (Fig. 3). They included 320, 417, 401, 214, 261, and 169 DAMs in T1 vs. CK, T3 vs. CK, T5 vs. CK, T3 vs. T1, T5 vs. T1, and T5 vs. T3 comparison groups, respectively (Fig. 3A, Table S1). Among the 569 DAMs, 20, including 19 flavonoids and one quinone were common in the six comparison groups (Fig. 3B). The 19 flavonoids were subdivided into 13 flavonols and six flavones, primarily quercetin-glycosides (7/19) (Table S1). Impressively, all 19 flavonoids were up-regulated in the comparison groups and were highly accumulated at T5 after topping (Fig. 3B), suggesting that these metabolites play an important role in axillary bud development. In addition, 37 DAMs, including 13 up-regulated and 24 down-regulated DAMs, were identified in the T1 vs. CK group compared to T3 vs. CK and T5 vs. CK groups (Fig. 3C). Among the 24 down-regulated DAMs, most of them had a fold-change range of two to four, except for IAA (pme1651), chrysoeriol-7-O-(6''-malonyl) glucoside (pmb0608), piperidine (pmb0782) and γ-glutamyl methionine (Zmdp001663), which were sharply decreased on T1 after topping (Table S1). Interestingly, levels of these four metabolites were largely normalized on T3 after topping (Figure S1), suggesting a link between altered metabolite levels on T1 and the axillary meristem initiation. There was also a significant positive correlation among these four metabolites, suggesting they may synergize during axillary meristem initiation (Figure S2).

**Figure 4:**
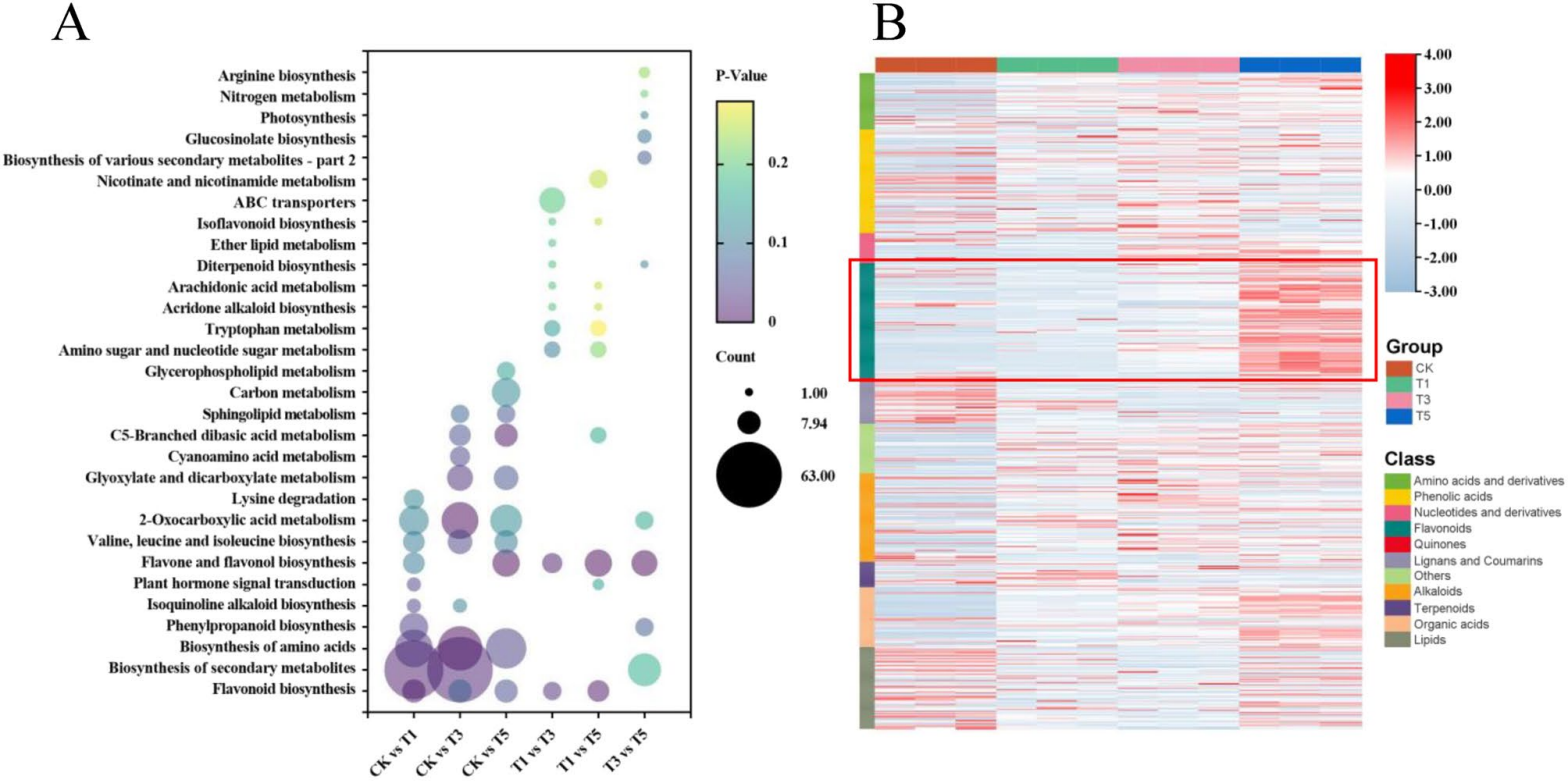
Flavonoid metabolism in the axillary bud after topping. (**A**) Top 10 enriched KEGG terms in each comparison group. (**B**) Hierarchical cluster analysis of all DAMs. The accumulation profiles of flavonoids were highlighted with the red box.

The top 10 enriched pathways based on KEGG enrichment analysis are shown in Fig. 4A. The DAMs in the T1 vs. CK, T3 vs. CK, and T5 vs. CK groups were enriched in the flavonoid biosynthesis, amino acids biosynthesis, and 2-Oxocarboxylic acid metabolism pathways. Interestingly, only the pathways related to flavonoid metabolism (flavonoid biosynthesis and flavone and flavonol biosynthesis) were significantly enriched (*p*-value < 0.05) by DAMs in T3 vs. T1, T5 vs. T1, and T5 vs. T3 comparison groups. A total of 100 differentially accumulated flavonoids were identified across all the comparison groups. These flavonoids accounted for the largest proportion (17.54%) of the DAMs (Table S1). Most of these flavonoids were up-regulated after topping and were highly accumulated at T5 (Fig. 4B), suggesting that the tobacco axillary bud has strong flavonoid biosynthesis activity after topping. Notably, flavonols and flavones accounted for 82% of all differentially accumulated flavonoids; most were identified in the glycosylated form (Table S1). These results revealed that flavonoid metabolism, especially related to flavonols and flavones, is essential in regulating axillary bud development.

### Comparative analysis of axillary bud proteome following topping

**Figure 5:**
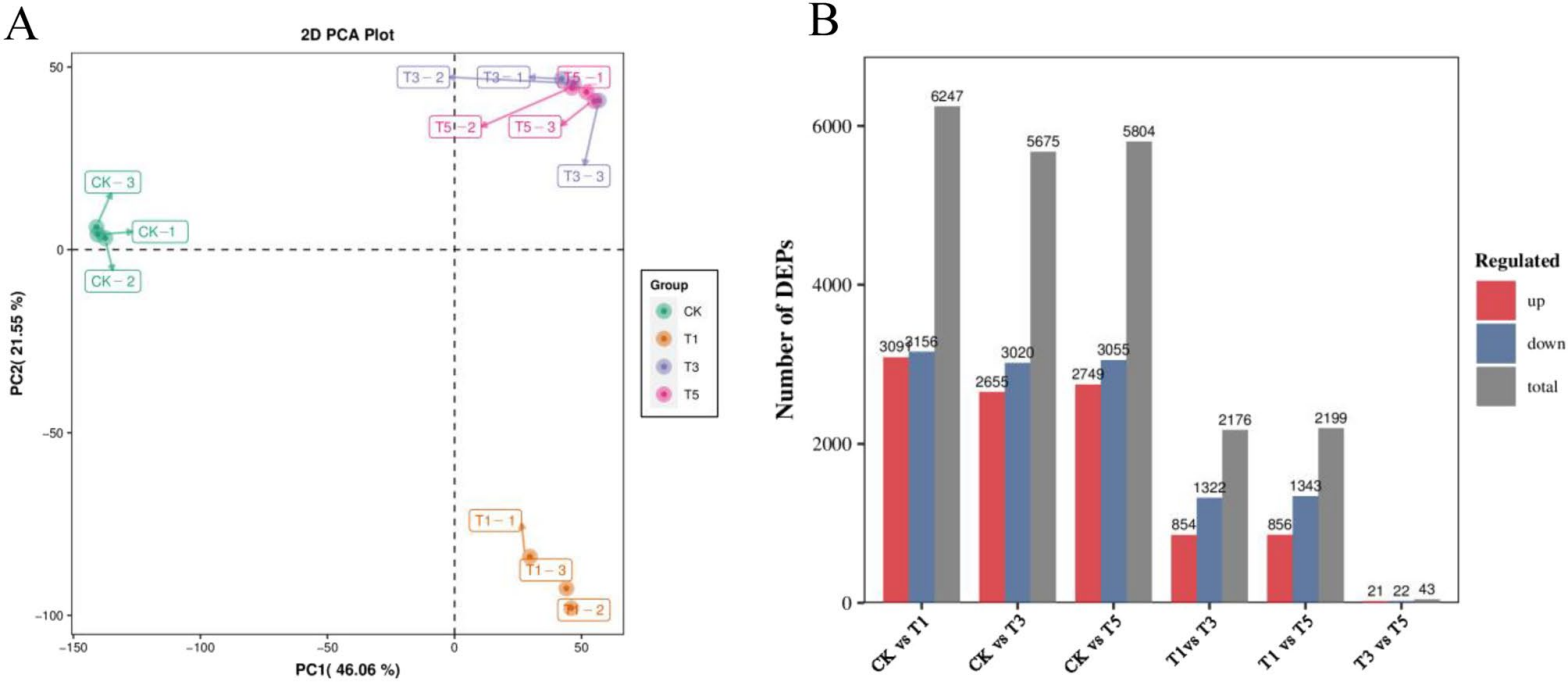
Proteome divergence among the four group samples. (**A**) PCA analysis plots of the four group samples. (**B**) DEPs statistics in six comparison groups.

A total of 15,486 credible proteins were identified and quantified by proteomics, and their temporal expression patterns were identified by PCA analysis. The CK group was differentiated from the T1, T3, and T5 groups on the PC1 axis and the T1 group from the T3 and T5 groups on the PC2 axis. Contrary to the metabolomics analysis, the T3 group was clustered with the T5 group, suggesting that the difference in protein expression profiles at these time points was limited (Fig. 5A).

Proteins with an FC > 1.5 or < 1/1.5 and *P* value < 0.05 were assigned as DEPs. A total of 9035 DEPs were identified from all comparison groups. The largest number of DEPs was detected between T1 and CK, where 6247 DEPs were identified, implying highly dynamic proteome changes within the axillary buds following topping. Additionally, 5675, 5804, 2176, and 2199 DEPs were detected in the T3 vs. CK, T5 vs. CK, T3 vs. T1, and T5 vs. T1 comparison groups, respectively. Only 43 DEPs were identified in the T5 vs. T3 comparison group (Fig. 5B).

**Figure 6:**
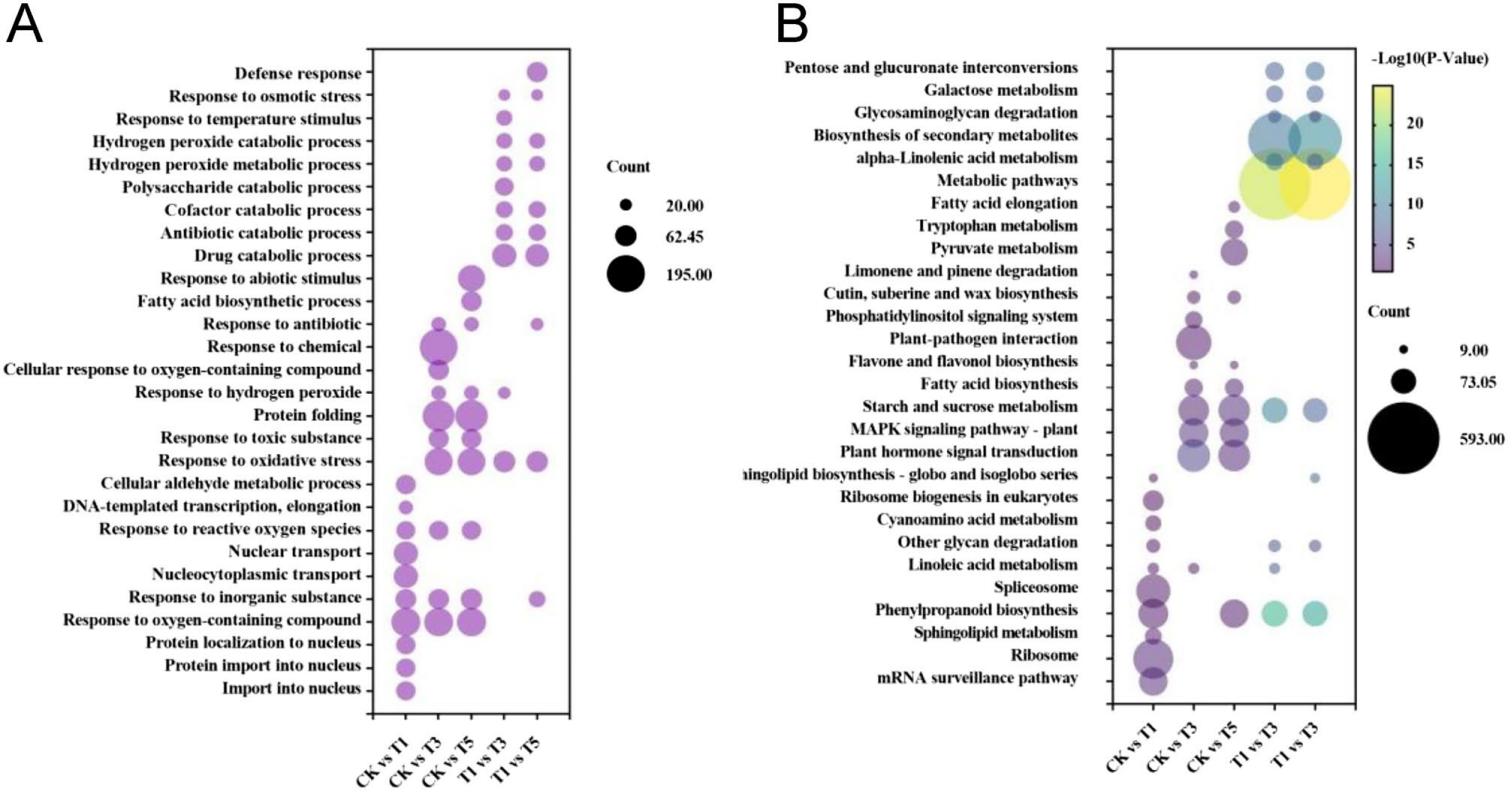
Enrichment analysis of DEPs in five comparison groups. (**A**) Top 10 enriched biological process GO terms in each comparison group. (**B**) Top 10 enriched KEGG terms in each comparison group.

Based on the GO enrichment analyses, all the DEPs were assigned to at least one of the three categories: biological process, cellular component, and molecular function. In the biological process, DEPs in all comparison groups were highly enriched in stimulus response-related processes, including response to oxygen-containing compounds, inorganic substances, and oxidative stress (Fig. 6A). In addition, the DEPs between T1 vs. CK were specifically enriched in transport-related processes, including the import into the nucleus, protein import into the nucleus, nucleocytoplasmic transport, and nuclear transport (Fig. 6A). KEGG enrichment analyses revealed that the DEPs in T1 vs. CK were mainly enriched in genetic information processing, consistent with the GO enrichment results. Additionally, the DEPs in T3 vs. CK and T5 vs. CK comparison groups were enriched in the plant hormone signal transduction, MAPK signaling pathway, and starch and sucrose metabolism pathways. At the same time, most DEPs in the T3 vs. T1 and T5 vs. T1 comparison groups were enriched in metabolic pathways and biosynthesis of secondary metabolites (Fig. 6B).

### Proteins related to the plant hormone signal transduction pathways following topping in tobacco

**Figure 7:**
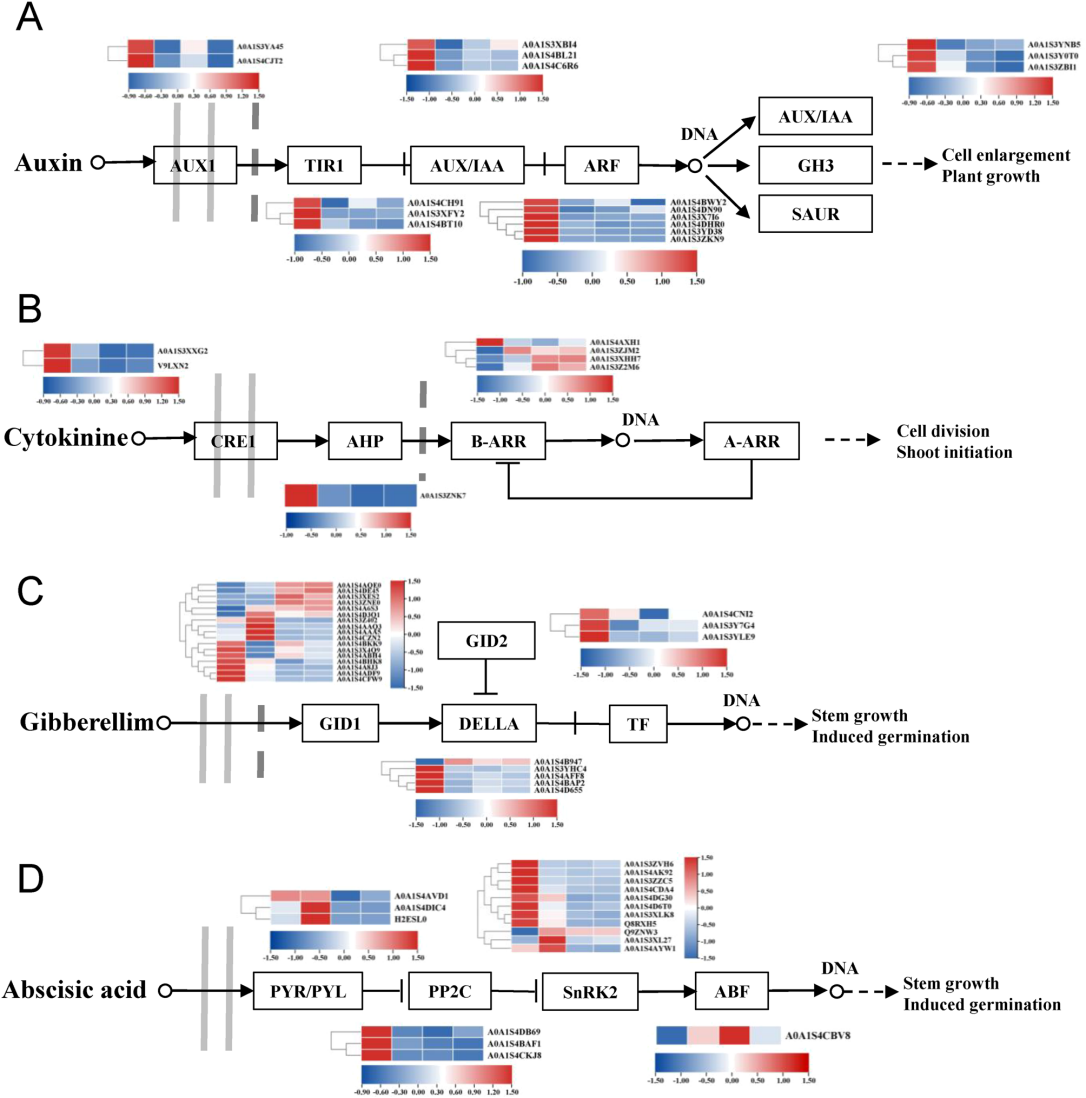
Expression patterns of the DEPs in the plant hormone signal transduction pathway. Auxin (**A**), Cytokinine (**B**), Gibberellin (**C**), and Abscisic acid (**D**) in the axillary bud proteomes of CK, T1, T3, and T5.

A total of 67 DEPs related to plant hormone signal transduction involving IAA, CTK, gibberellin, and abscisic acid (ABA) were identified (Fig. 7, Table S2). Among them, 17 DEPs were involved in IAA signal transduction including auxin transporter protein, transport inhibitor response 1 (TIR1), auxin-responsive protein (AUX/IAA), auxin response factor (ARF), and the indole-3-acetic acid-amido synthetase. Almost all of them were significantly down-regulated after topping, except AUX/IAA, which was up-regulated in T5 (Fig. 7A). For the cytokinin signal transduction pathway, seven DEPs were identified, including two histidine kinases (CRE1), one histidine-containing phosphotransferase protein (AHP), and four two-component response regulators (B-ARR). CRE1, AHP, and one B-ARR expression were down-regulated in T1, T3, and T5. On the contrary, the other three B-ARRs were up-regulated after topping (Fig. 7B). Additionally, the 25 DEPs involved in the gibberellin signal transduction pathways showed a complex expression pattern, which was particularly obvious with gibberellin-insensitive dwarf 1(GID1) (Fig. 7C). There were 7, 4, and 4 GID1 were up-regulated in T1 vs. CK, T3 vs. CK, T5 vs. CK, respectively. At the same time, there were 6, 4, and 4 GID1 down-regulated in T1 vs. CK, T3 vs. CK, T5 vs. CK, respectively. Among them, 3 and 2 GIDs were up-regulated and down-regulated in the three comparison groups, respectively (Fig. 7C). Besides, the DEPs involved in ABA signal transduction pathways had similar expression patterns with those involved in cytokinin signaling. Most of these related proteins were down-regulated after topping (Fig. 7D).

### Proteins related to redox homeostasis following topping in tobacco

Bud outgrowth is regulated by redox homeostasis in plants^18^. Antioxidant enzymes play a major role in the maintenance of cellular redox homeostasis. A total of 87 proteins involved in redox homeostasis, including 2 glutathione reductase (GR), 2 NAD(P)H-quinone oxidoreductase, 5 catalase (CAT), 5 monodehydroascorbate reductase (MDAR), 9 superoxide dismutase (SOD), 15 glutathione S-transferase (GST), and 49 peroxidase (POD) were differentially expressed after topping compared to CK (Figure S4, Table S3). Among them, 44, 34, and 30 DEPs were up-regulated in T1 vs. CK, T3 vs. CK and T5 vs. CK comparison groups, respectively (Table S3). At the same time, 14, 27, and 26 DEPs were down-regulated in T1 vs. CK, T3 vs. CK and T5 vs. CK comparison groups, respectively (Table S3). Additionally, 22 and 12 DEPs were up-regulated and down-regulated in all three comparison groups, respectively (Table S3). Specifically, the expression level of 21 proteins, including 2 MDAR, 2 SOD, 7 GST, and 10 POD, were up-regulated at T1 and reverted to normal at T3 and T5 compared to CK (Table S3).

### Proteins involved in flavonols and flavones biosynthesis following topping in tobacco

**Figure 8:**
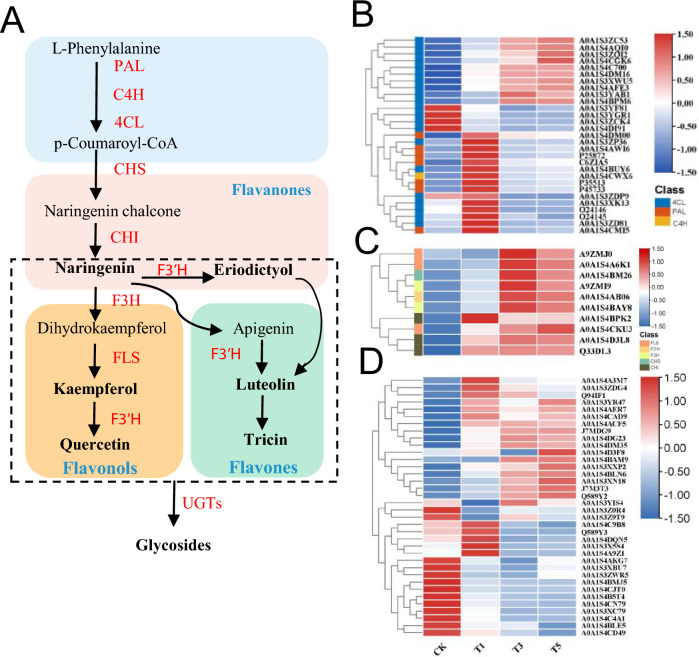
Expression patterns of the DEPs involved in flavonols and flavones biosynthesis. (**A**) Flavonols and flavones biosynthesis pathway in tobacco. The proteins identified in the proteome are shown in red, and the main flavonoid products in the metabolome are shown in bold. (**B**, **C**) Heat map of DEPs expression patterns during flavonols and flavones biosynthesis. (**D**) Heat map of UGTs differential expression patterns after topping.

Besides the antioxidant enzymes, non-enzymatic antioxidants, such as flavonoids, also play an important role in redox homeostasis. Considering the wide variations in flavonols and flavones in the axillary bud after topping, this study focused on DEPs associated with the biosynthesis of these two compounds (Fig. 8A). Flavonoid biosynthesis takes place through the phenylpropanoid pathway, catalyzed by three enzymes, including phenylalanine ammonia-lyase (PAL), trans-cinnamate 4-monooxy-genase (C4H), and 4-coumarate–CoA ligase (4CL). These enzymes catalyze the formation of p-coumaroyl-CoA. Herein, 29 DEPs, including 7 PAL, 1 C4H, and 21 4CL, were involved in p-coumaroyl-CoA synthesis (Fig. 8B). Among them, 10 4CL were up-regulated during axillary bud development after topping, and four were down-regulated. Additionally, the expression level of 11 proteins, including 5 PAL, 6 4CL, and 1 C4H, were up-regulated at T1 and reverted to normal at T3 and T5 compared to CK (Table S4). Key enzymes catalyzed the formation of flavonols and flavones from p-coumaroyl CoA (Fig. 8A). These key enzymes comprised 10 DEPs, including 1 chalcone synthase (CHS), 3 chalcone isomerase (CHI), 2 flavanone 3-hydroxylase (F3H), 3 flavonol synthase (FLS), and 1 flavonoid 3’-hydroxylase (F3’H) (Fig. 8C). Interestingly, they were up-regulated during axillary bud development after topping (Table S4). Additionally, 36 UDP-glycosyltransferase (UGT), which catalyzes the synthesis of galactosylated derivatives from various substrates, and a key modifying enzyme in flavonoid metabolism were differentially expressed after topping compared to CK (Fig. 8D). The up-regulated UGTs in T1 vs. CK, T3 vs. CK, and T5 vs. CK were 17, 11, and 13, respectively (Table S4). At the same time, the down-regulated UGTs in T1 vs. CK, T3 vs. CK, and T5 vs. CK were 9, 12, and 9, respectively (Table S4). Among them, 7 and 3 UGTs were up-regulated and down-regulated in the three comparison groups (Table S4). In summary, many proteins involved in the flavonoid pathway were significantly regulated during axillary bud development, consistent with the change in flavonoid content in axillary buds after topping.

### Correlations between the metabolome and proteome during axillary bud development in tobacco

**Figure 9:**
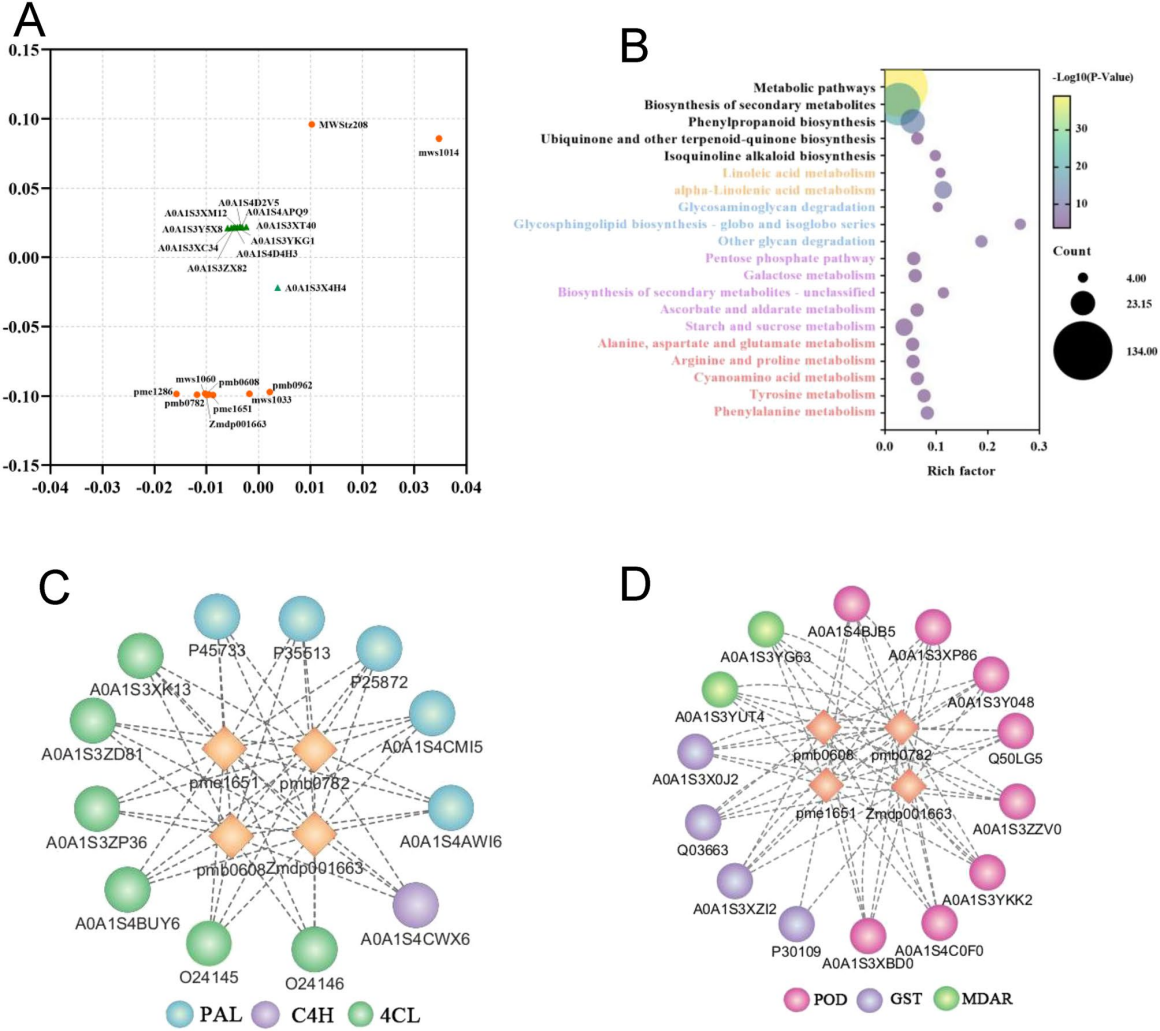
Joint metabolome and proteome analysis. (**A**) The top 10 influential metabolites and proteins based on O2PLS analysis. (**B**) The top 20 enriched KEGG terms of 435 DEPs. (**C**, **D**) Correlation network analysis between DEPs and metabolites. Metabolites and proteins are indicated as rhombus and circles, respectively.

The top 10 metabolites and proteins with the most influence on the model based on O2PLS are shown in Fig. 9A. The proteins with the highest influence on the metabolome were involved in carbohydrate metabolic, oxidation–reduction, and genetic information processes (Table S5). The top 10 metabolites included 3 amino acid derivatives, 3 alkaloids, 2 flavonoids, 1 nucleotide derivative, and 1 coumarin. IAA was among the most influential metabolites that regulate axillary bud development. Notably, chrysoeriol-7-O-(6''-malonyl)-glucoside, piperidine, and γ-glutamyl methionine were significantly positively correlated with IAA and were among the most influential metabolites (Table S5).

Correlation analysis between the four metabolites (IAA, chrysoeriol-7-O-(6''-malonyl)-glucoside, piperidine, and γ-glutamyl methionine) and the DEPs revealed that 476 DEPs were significantly correlated with the four metabolites (Table S6). Among them, 435 DEPs were negatively correlated and were involved in metabolic pathways, such as phenylpropanoid biosynthesis, carbohydrate metabolism, amino acid metabolism, and lipid metabolism (Fig. 9B). The other 41 DEPs were positively correlated with the four metabolites and were involved in RNA transport and DNA replication (Table S6). In the phenylpropanoid biosynthesis pathway, 13 proteins (5 PAL, 6 4CL, and 1 C4H) negatively correlated with the four metabolites were involved in p-coumaroyl-CoA synthesis (Fig. 9C), suggesting that the accumulation of these metabolites had a negative effect on synthesis of p-coumaroyl-CoA. Besides, in the metabolic pathways, 14 ROS-related DEPs, including 8 POD, 2 MDAR, and 4 GST, were negatively correlated with the four metabolites (Fig. 9D). These results imply that IAA and three other metabolites (chrysoeriol-7-O-(6''-malonyl)-glucoside, piperidine, and γ-glutamyl methionine) regulate axillary bud development through multiple metabolic pathways and influence redox homeostasis.

## Discussion

Topping is a necessary agronomic practice in tobacco. It alters various biological processes by switching the plant from its reproductive to its vegetative phase. Subsequently, this alters nicotine biosynthesis, the hormonal balance, root development, and the source-sink relationship^19–21^. The elongation of axillary buds is a typical plant response to topping. This phenomenon was also observed in our study, where the length of axillary buds increased rapidly after topping (Fig. 1). Omics technology can provide a more comprehensive perspective for understanding of the molecular mechanisms underlying axillary bud growth in response to topping. Previous research have revealed that the growth of axillary bud may be controlled by genes involved in carbohydrate metabolism and in the hormone signal transduction pathway^14^. Consistent with this view, our proteome data showed that DEPs in T3 vs. CK and T5 vs. CK were mainly enriched in plant hormone signal transduction, MAPK signaling pathway, and starch and sucrose metabolism (Fig. 6B). Many phytohormones, including IAA, CTK, gibberellin, and ABA, regulate the axillary bud development and can influence each other in various ways^12,22–24^. IAA is produced by the shoot apex and transported basipetally through the stem, and inhibits growth of axillary bud in indirect ways since it does not enter the buds^7^. The IAA depletion in the main stem following topping control bud outgrowth by inhibiting SL and inducing CTK^8,9^. Both CTK and SL can move into the buds, and control the bud outgrowth by regulating, at least partly, the expression of the the transcription factor *BRC1*^25^. Gibberellin and ABA are also involved in axillary bud development. However, their role is less investigated than the roles of IAA, CTK, and SL^26–28^. In the present study, 67 DEPs related to these hormone signal transduction were identified, with most of them down-regulated after topping (Fig. 7, Table S2), consistent with the findings from previous studies wherein the majority of the hormone-related genes were down-regulated after topping^14^.

On the other hand, IAA depletion from the main stem following topping allows canalization driven IAA export from axillary buds, and this is hypothesized to be required for bud outgrowth^29–32^. Based on metabolome results, we found the IAA content in axillary buds decreased sharply at day one after topping (Figure S1, Table S1), which was probably caused by IAA export from buds. To date, there is a lot of evidences that the growth arrest of bud often correlate with the elevated IAA content and strong IAA signaling in buds^11,33^. For example, the expression of *AUX/IAA* and *SAUF* genes were up-regulated in dormant buds of strawberry^34^. Additionally, most of IAA signaling relevent genes were down-regulated in growing buds of tobacco after topping^14^. Consistently, we also identified several IAA signaling relevent proteins, such as ARF, AUX/IAA, and TIR1, were down-regulated at T1, T3, and T5 in topped plant (Fig. 7). These results suggested that reduction of IAA levels and inhibition of IAA signal transduction were associated with axillary buds’ release from dormancy in tobacco following topping. It is noted that the concentration of IAA in buds of topped plant returned to similar levels as controls (CK) at day three and remained stable until day five (Figure S1, Table S1). Several studies have confirmed that the upper axillary buds will become a new source of IAA to restore apical dominance in topped plant, and the sustained growth of buds require both IAA synthesis and transport^32,35^. Therefore, our results suggested that IAA alteration in buds might play a critical role in dormancy release and sustained growth of axillary bud.

However, the role of the concentration variance of IAA in axillary bud outgrowth is not yet clear. In the present study, we identified many DEPs (476) strongly correlated with IAA contents, including 435 negatively correlated DEPs, which were mainly enriched in carbohydrate, amino acid, and lipid metabolism (Fig. 9, Table S6). Carbohydrate metabolism is involved in many essential processes in plants, as it is a energy source, components of natural architecture and is involved in the transfer of information^16,36^. Evidence is emerging that sucrose and trehalose 6-phosphate play a central role in the control of bud release^24^. Lipid signaling molecules are also involved in axillary bud growth. For example, *BnFAX6* mediating fatty acid export from plastids functions in axillary bud dormancy release, possibly through enhancing linoleic acid level in axillary buds of *Brassica napus*^37^. Amino acids are primary units of proteins, and also serve as energy sources, chemical messengers and precursors of diverse metabolites^38^. Therefore, amino acid metabolism play a fundamental role in plant growth and development. Taken together, our results provide clues for further research on whether IAA in axillary buds regulates bud development by influencing the expression level of proteins associated with these metabolic pathways. Additionally, we also identified another three metabolites, including piperidine, γ-glutamyl methionine and chrysoeriol-7-O-(6''-malonyl) glucoside, which seemed had a synergistic role with IAA in regulating the axillary bud development. All of them were among the most influential metabolites in the O2PLS model, and showed a similar change profile following topping and significantly correlated with the 476 DEPs (Fig. 9A, Figure S2, Table S6). However, how these metabolites interact with IAA to affect axillary bud development remained unclear. Herein, we suggested that these metabolites possibly control buds outgrowth by regulating IAA transport. Chrysoeriol-7-O-(6'-malonyl) glucoside is a flavonoid, this kind of substances have a similar structure to the auxin inhibitor N-1-naphthylphthalamic acid and can help accumulate IAA by inhibiting IAA polar transport^39^. In *Arabidopsis*, a change in flavonoid profile, level and/or distribution can lead to redistribution of auxin efflux carriers, and results in changing of IAA levels^40^. Current studies have also indicates that glutathione can alter IAA transport and metabolism, and its re-synthesis through the γ-glutamyl cycle requires the participation of γ-glutamyl methionine^41,42^. Therefore, it is possible that the down-regulation of these metabolites induced by topping enhanced the IAA polar transport and then reduced the content of IAA in buds. If this was the case, functional studies of these substances may provide more options for screening natural inhibitors to control axillary bud outgrowth.

In this study, we also found a striking enrichment of metabolites and proteins related to flavonoid biosynthesis and metabolism (Figure S3, Fig. 8). Flavonoids are secondary metabolites widely found in plants, which are involved in various processes of plant growth, such as axillary buds outgrowth, male fertility, root development, and pigmentation^43,44^. Previous observations indicate that a high level of flavonoid and flavonoid pathway genes can repress the outgrowth of axillary buds by inhibiting the IAA transport^10^. On the contrary, most of flavonoid and flavonoid pathway genes identified from our omics data were up-regulated in growing buds. Additionally, several DEPs involved in p-coumaroyl-CoA synthesis, which is a common precursor of flavonoids synthesis, showed a negative correlation with the IAA content (Fig. 8C). Therefore, if the mechanism of flavonoid-dependent axillary bud inhibition also applicable to tobacco, it is likely that only a few flavonoids are responsible for the regulation of IAA transport, such as chrysoeriol-7-O-(6'-malonyl) glucoside. On the other hand, accumulation of flavonoids in buds after topping might have another functions. Flavonoids can protect plants against biotic and abiotic stressors, such as herbivores, bacteria, fungi, and ultraviolet^45,46^. Topping is a form of mechanical damage sustained at the aboveground plant part, which can lead to the accumulation of ROS, including H_2_O_2_, superoxide anion, and hydroxyl radicals, subsequently inducing oxidative stress to the plant^47–50^. Under stress, plants trigger their oxidative defense system to balance ROS synthesis and scavenging by increasing the levels/activities of enzymatic and non-enzymatic antioxidants. Flavonoids can act as non-enzymatic antioxidants to reduce ROS levels by direct ROS scavenging, inhibiting the activities of free radical-generating enzymes, and activating enzymatic antioxidants^45^. Besides flavonoids, we also identified large number of enzymatic antioxidants, such as SOD, POD, GST, CAT, and GR, and most of which were up-regulated after topping (Figure S4, Table S3). Among them, several enzymatic antioxidants were negatively correlated with the IAA content (Fig. 9D). Several studies have revealed that stress-induced ROS can alter the IAA gradient and interfere with IAA-mediated signal transduction. In turn, IAA induces ROS production and regulates its homeostasis by influencing antioxidant levels^51^. Anyway, there seems to be an interesting crosstalk of IAA alteration and ROS homeostasis in axillary bud outgrowth of tobacco under topping stress.

## Conclusion

A total of 9035 DEPs and 569 DAMs were identified from proteomic and metabolomic data of the axillary bud following topping in tobacco. Our work highlight the role of IAA alteration in axillary bud outgrowth, and implied a potential crosstalk among IAA alteration, flavonoids accumulation profile and ROS homeostasis. Additionally, three metabolites (piperidine, γ-glutamyl methionine and chrysoeriol-7-O-(6''-malonyl) glucoside) were newly identified from axillary buds in tobacco, which might act as inhibitor of IAA transport. The findings in this study will substantially expand the current spectrum of how to define the axillary bud at the proteomic and metabolomic levels, providing valuable metabolites coupled with proteins for further functional characterization.

## Materials and methods

### Plant materials and cultivation

K326, the main tobacco cultivar in China, characterized by strong axillary growth, was used as the model crop in this study. The use of plants in study complies with international, national and/or institutional guidelines. K326 seeds were germinated in a mixture of 60% (w/w) peat culture substrate, 20% (w/w) ground maize stalk, and 20% (w/w) perlite in a naturally illuminated glasshouse. Two months from sowing, the seedlings were transferred into 4 L pots (one plant per pot) containing paddy soil. The plants were watered daily.

Five weeks after transplanting, the head and two leaves below the flag leaf were topped. The upper axillary buds were collected before and 1, 3, and 5 days after topping. Upper axillary buds from seven individual tobacco plants were collected as a biological replicate and three biological replicates were harvested^66^, giving a total of 12 samples. Samples were immediately frozen in liquid nitrogen and stored at − 80℃, awaiting further analysis.

### Metabolomics and differentially accumulated metabolite (DAM) analysis

The sample preparation and metabolome profiling were performed by Metware Biotechnology Co., Ltd. (Wuhan, China) following their standard procedures. All samples were freeze-dried using a vacuum freeze-dryer (Scientz-100F) and then ground into powders. Dissolve 50 mg of powder with 1.2 mL 70% methanol solution, vortex 30 s every 30 min for 6 times in total. Following centrifugation at 12,000 rpm for 3 min, the extracts were filtrated before UPLC-MS/MS analysis. All sample extracts were mixed in equal proportions to generate samples for quality control.

The sample extracts were analyzed using an UPLC system (SHIMADZU Nexera X2) coupled with tandem mass spectrometry (MS/MS, Applied Biosystems 4500 Q TRAP), running in electrospray ionization mode. The column in the UPLC system was Agilent SB-C18 (1.8 µm, 2.1 mm × 100 mm). The mobile phase was consisted of solvent A (pure water with 0.1% formic acid) and solvent B (acetonitrile with 0.1% formic acid). Sample measurements were performed with a gradient program that employed the starting conditions of 95% A, 5% B. Within 9 min, a linear gradient to 5% A, 95% B was programmed, and a composition of 5% A, 95% B was kept for 1 min. Subsequently, a composition of 95% A, 5.0% B was adjusted within 1.1 min and kept for 2.9 min. The flow velocity was set as 0.35 mL per minute; The column oven was set to 40 °C and the injection volume was 4 μL. The ESI source operation parameters were as follows: source temperature 550 °C; ion spray voltage 5500 V (positive ion mode)/− 4500 V (negative ion mode); ion source gas I, gas II, curtain gas were set at 50, 60, and 25 psi, respectively; the collision-activated dissociation was high.

Analyst 1.6.3 software was used for data filtering, peak detection, alignment and calculations. Qualitative analysis was performed base on the Metware Database, the substance was characterized according to the secondary spectral information. Metabolite quantification was conducted through multiple reaction monitoring analysis using triple quadrupole mass spectrometry. Unsupervised principal component analysis (PCA) and orthogonal partial least squares discriminant analysis (OPLS-DA) were carried out with the package MetaboAnalystR. The variable importance in projection (VIP) score was obtained by the OPLS-DA model, and metabolites with VIP ≥ 1, absolute log2 fold change (FC) ≥ 1, and *p*-value ≤ 0.05 were defined as DAMs. Hierarchical cluster analysis and higher Venn diagram were visualized by TBtool v2.080. Moreover, R software analyzed Pearson correlation coefficients between treatments and metabolites. The DAM functions were characterized using GO/KEGG enrichment analyses.

### Proteomics and DEP analysis

The proteins in the 12 samples were quantified using the BCA Kit (P0010S) according to the manufacturer’s instructions. Half of each sample was used for SDS-PAGE detection and to determine the protein concentration, while the other half was used for trypsin hydrolysis. After desalting the enzymatic hydrolysis peptide, the proteins in the samples were analyzed by LC–MS/MS.

Liquid chromatography (LC) was performed on a nanoElute UHPLC (Bruker Daltonics, Germany). About 200 ng peptides were separated within 60 min on a commercially available reverse-phase C18 column with an integrated CaptiveSpray Emitter (25 cm × 75 μm, IonOpticks, Australia). The gradient was run at 300 nL/min starting from 2 to 22% of solvent B in 45 min, then going up to 35% in 5 min, then going up to 80% in 5 min, and finally maintenance at 80% B for 5 min.

The LC was coupled online to a hybrid timsTOF Pro2 (Bruker Daltonics, Germany) via a CaptiveSpray nano-electrospray ion source. To establish the applicable acquisition windws for DIA-PASEF mode, the timsTOF Pro2 was operated in DDA-PASEF mode with 10 MS/MS frames in 1 complete frame. The capillary voltage was set to 1400 V, and the MS and MS/MS spectra were acquired from 100 to 1700 m/z. As for ion mobility range (1/K0), 0.7 to 1.4 Vs/cm2 was used. The TIMS accumulation and ramp time were both set to 100 ms, which enable an operation at duty cycles close to 100%.

The raw MS/MS data were analyzed using DIA-NN (v1.8) against uniprot-proteome_UP000084051_yancao_20220719.fasta database (73,608 entries). The false discovery rate (FDR) of search results was adjusted to < 1% at both protein and precursor ion levels, the remaining identifications were used for further quantification analysis. Protein quantification was analyzed by MaxLFQ method. The discovered protein annotation information was retrieved using UniProt, KEGG, GO, KOG/COG, and other databases to mine the protein functions. Two standards (based on trustworthy proteins) were chosen to determine the difference between samples. A minimum FC of 1.5 and *p*-value < 0.05 were selected as the screening criteria to distinguish a DEP. Finally, DEP functions were characterized by GO/KEGG enrichment analyses. The datasets used and/or analysed during the current study available from the corresponding author on reasonable request.

### Joint metabolome and proteome analysis

Integration analysis was performed by two-way orthogonal partial least squares (O2PLS) to identify the differentially expressed metabolites and proteins. Only the pairs with a Pearson correlation coefficient > 0.9 or < − 0.9 and *P*-value < 0.05 were included in the analysis.

### Supplementary Information

Supplementary Figures.

Supplementary Table 2.

Supplementary Table 3.

Supplementary Table 4.

Supplementary Table 5.

Supplementary Table 6.

Supplementary Table 7.

Supplementary Legends.

## Data Availability

The datasets used and/or analysed during the current study available from the corresponding author on reasonable request.
