# Supplementary material for: Integrative proteome and metabolome unveil the central role of IAA alteration in axillary bud development following topping in tobacco
Source: Sci Rep. 2024 Jul 3;14:15309. doi: 10.1038/s41598-024-66136-4 (PMC11222511; doi:10.1038/s41598-024-66136-4)

Supplementary Figure S1

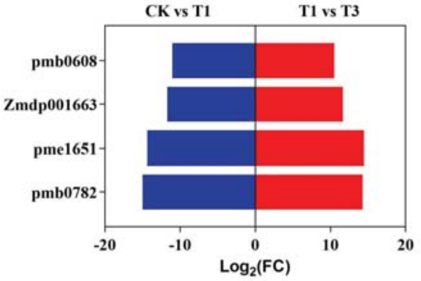

Supplementary Figure S2

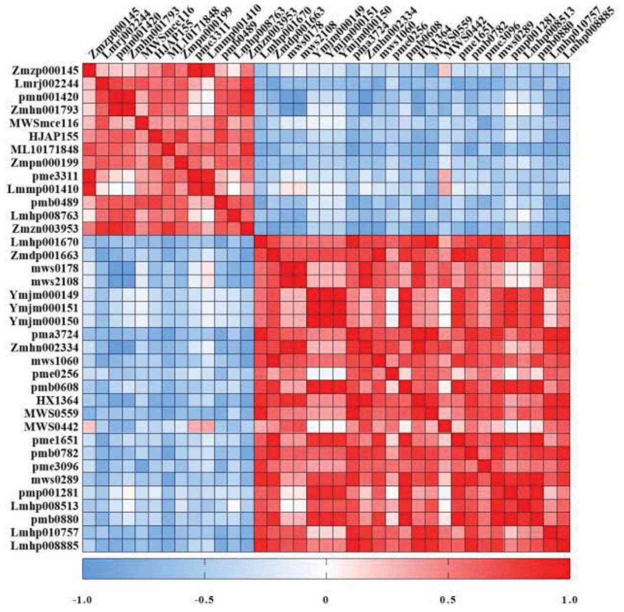

Supplementary Figure S3

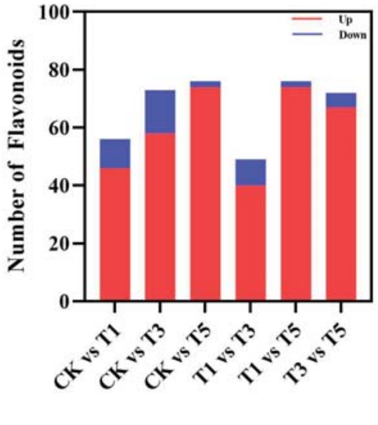

Supplementary Figure S4

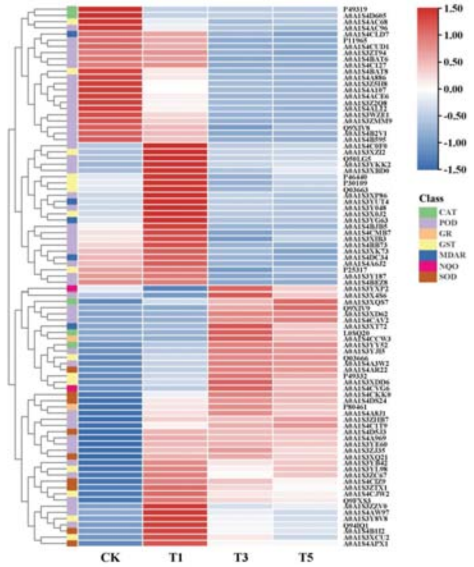

Supplement: Supplementary file 1 — Supplementary Figures. [file 41598_2024_66136_MOESM1_ESM.pdf]
