# Supplementary material for: Integrative proteome and metabolome unveil the central role of IAA alteration in axillary bud development following topping in tobacco
Source: Sci Rep. 2024 Jul 3;14:15309. doi: 10.1038/s41598-024-66136-4 (PMC11222511; doi:10.1038/s41598-024-66136-4)
Supplement: Supplementary file 8 — Supplementary Legends. [file 41598_2024_66136_MOESM8_ESM.docx]

**Supplementary information**

**Table S1** Summary of differentially accumulated metabolites.

**Table S2** List of differentially expressed proteins related to plant hormones.

**Table S3** List of differentially expressed proteins related to redox homeostasis.

**Table S4** List of differentially expressed proteins related to flavonoid synthesis.

**Table S5** O2PLS analysis between proteome and metabolome.

**Table S6** Correlation network analysis between DEPs and IAA signal transduction.

**Figure S1** Dynamic changes of four metabolites in CK vs. T1 (blue) and T1 vs. T3 (red) comparison groups.

**Figure S2** Correlation heatmap of 37 metabolites.

**Figure S3** The differentially accumulated flavonoid statistics for each comparison group.

The up-regulated flavonoids are located in the red regions and the down-regulated flavonoids in the blue regions.

**Figure S4** Expression patterns of the DEPs involved in ROS scavenging.
